# Configurational Entropy Relaxation of Silica Glass—Molecular Dynamics Simulations

**DOI:** 10.3390/e23070885

**Published:** 2021-07-13

**Authors:** Ondrej Gedeon

**Affiliations:** Department of Glass and Ceramics, University of Chemistry and Technology Prague, Technicka 5, CZ-166 28 Prague, Czech Republic; Ondrej.Gedeon@vscht.cz

**Keywords:** configurational entropy, glass transition, molecular dynamics, silica glass

## Abstract

Vitreous silica was modelled using molecular dynamics (MD). The glass structure was transferred into an undirected graph and decomposed into disjoint structural units that were ideally mixed to calculate the configurational entropy. The Debye relaxation model was suggested to simulate the evolution of entropy during the cooling of the system. It was found that the relaxation of the configurational entropy of MD corresponds to the effective cooling rate of 6.3 × 10^6^ Ks^−1^ and its extrapolation to 0.33 Ks^−1^ mimics the glass transition with *T_g_*; close to the experimental value. Debye relaxation correctly describes the observed MD evolution of configurational entropy and explains the existence of freezing-in temperature and the shape of the curve in the transition region.

## 1. Introduction

Molecular dynamics (MD) mimics glass formation despite the effective cooling rates being many orders higher compared to the real glass quenching. Atomistic simulations must cope with two opposite requirements: (1) the demand for a sufficiently high number of atoms in order to not miss any important structural feature and to obtain a reliable statistical distribution, and (2) the need for a high precision description of the atomic interaction (ideally using quantum ab initio calculations). MD with empirical potentials meets the first goal and generates a virtual structure comparable to real glass. MD provides a wealth of information, especially about the glass structure that is experimentally unattainable or limited to specific structural patterns. In contrast to experiments, MD offers a detailed picture of a time-evolution of the studied mechanical system that can be further processed using powerful tools of statistical physics to obtain macroscopic variables directly comparable with experimental data. One of the main obstacles of the transition from statistical mechanics to thermodynamics is the evaluation of entropy, especially configurational entropy, which, as is widely believed in the glass community, plays a decisive role in the solidification of melts [1,2,3,4,5,6,7]. Determination of configurational entropy from the definition is an unreachable task due to the need for enormous computation power; therefore, models must be constructed instead. A promising approach is basic ring statistics [7]; the method yields the correct specific heat step at *T_g_* for vitreous silica and correctly describes the cooling–heating relaxation effects in the glass transition region [8]. The connection between configurational entropy and structure was also revealed, and the structural origin of the Vogel temperature as the temperature of effective freezing-in of the structure was also suggested [9]. Nevertheless, the model was based on empirical findings only, and the found quadratic dependence of the configurational entropy on the temperature in the glass transition region was neither explained nor physically described.

In the present paper, the previous ideas are extended to comprehend the observed behaviour of configurational entropy of MD glass in the glass transition region. Furthermore, the configurational entropy obtained from MD simulation will be compared with the Debye relaxation of entropy during the cooling procedure. It should show if, and by how much, the simple Debye relaxation deviates from the expected, more complex relaxation in the transition region. In addition, solid reasoning and understanding can be established for the empirical findings presented in previous papers.

## 2. Materials and Methods

### 2.1. Molecular Dynamics

Classical MD with BKS [10] potentials was used to model the vitreous SiO_2_-system. The details of the simulation can be found in [11]. The simulation comprises 7200 atoms (2400 Si and 4800 O) and utilises DL_Poly code [12]. The system was kept under constant pressure (NpE ensemble). The simulation started at 5000 K by mixing and equilibrating the system for 25 ps. The cooling procedure down to 100 K consists of 100 deg consecutive temperature steps; each step comprises the numeric control of the adjusted temperature for 2500 time steps followed by NpE simulation for the next 7500 time steps. Integration time step set to 1 fs resulted in the effective cooling rate of 10^13^ Ks^−1^.

In total, 150 consecutive snapshots of the structure with 0.15 ps time-step were recorded at each temperature. The structures at each temperature were transformed into undirected graphs and processed as shown below.

The transformation of the atomic positions (geometrical representation of structure) into a graph (topological representation) is carried out in the following way. Two silicon atoms are connected by an edge (link) if an oxygen atom (bridging oxygen) provides a bond between them. The existence of the bond is based on the distance criterion (details can be found in [6]). A graph is the set of vertices (silicon atoms) and edges (bridging oxygen atoms). Since the edges are symmetric (no preference of vertex), the graph is undirected.

The medium-range order of glass can be described by the topological descriptors; among them rings (closed paths along the edges of the graph) are the most often used. However, there the above definition is too wide as it generates a large number of overlapping rings. More restrictive definitions of the rings are utilised to decrease the number of rings in the glass structure. (1) Primitive ring [13] (or minimal ring [14]) is a ring that cannot be written as a sum of two shorter rings, (2) strong ring [15] is a ring that is not the sum of smaller rings (note “two” is omitted in comparison to the primitive ring), and (3) very strong ring is a ring that contains at least one edge not belonging to a shorter ring. None of the definitions are free from redundancy because one vertex/edge can be a member of a few rings/strong rings/very strong rings [15]. It means the structure cannot be decomposed into disjoint units and an even more restrictive definition of the ring is needed. Hence, the basic rings were introduced [7]. In the last definition, a vertex can be a part of only the smallest ring of all, of which it is a member.

### 2.2. Configurational Entropy

The configurational entropy can be calculated, from the definition, as the logarithm of the number of different configurations at the given energy. This task, however, is impossible to perform for a large ensemble. An alternative approach to evaluate the configurational entropy based on the decomposition of the glass structure into disjunctive units called basic rings has been suggested [7]. The idea consists of the explicit inclusion of the rings in decomposing the glass network so that the medium-range order is preserved in a specific way. The decomposition into basic rings, contrary to the often-used primitive rings, generates disjunctive pieces of structure that can be directly used for the calculation of the configurational entropy, *S.* Random mixing of the structural units obtained by the decomposition yields entropy

(1) $$S=-\frac{kN_{A}}{N_{Si}}\left[M\sum_{\left(j,k\right)}^{m_{C}}r\left(j,k\right)\ln r\left(j,k\right)+N_{O}\sum_{i}^{m_{O}}q_{i}\ln q_{i}\right],$$

where *N_Si_* is the number of the silicon atoms (vertices in the graph), *M* is the total number of the determined structural units consisting of at least one vertex, *N_O_* is the total number of residual oxygen atoms (not included in the previous Si-containing units), *N_A_* is the Avogadro constant; *k* is the Boltzmann constant, *m_C_* is the total number of different Si-containing structural units with the corresponding statistical weight *r*(*j*,*k*); the sum runs over (*j*,*k*) pairs, distinguishing the units by either length *j* or size *k*; and *m_O_* is the number of different oxygen atoms (*i* runs over bridging, non-bridging, over coordinated, and free oxygen atoms). Each type of oxygen is present with the corresponding statistical weight *q_i_*. The length of a unit equals the number of Si in the basic ring; in units not comprising a ring, the length is identical to the number of Si. The size of the unit is defined as the total number of Si in the unit. In simple units, not consisting of more than one basic ring, the length and size coincide. It should be noted that the approach does not provide a complete classification of the topologically different units. Complex units not differentiated by this algorithm are very rare; therefore, the simplified classification does not influence the entropy evaluation.

### 2.3. Entropy Relaxation

The ensemble corresponding to the cooled-down melt is permanently out of equilibrium due to the continuous cooling. Nevertheless, the difference between the equilibrium and the actual states is undistinguishable at high temperatures. The path of the (actual) ensemble during the cooling is assumed to be parametrised by the corresponding path of the equilibrated ensemble, represented by the state variable(s) ξ. We distinguish between the actual configurational entropy of the system at time *t*, *S*(*t*)*,* and that corresponding to the equilibrium state described by the variables ξ, $\tilde{S}\left(\xi \left(t\right)\right)$. Relaxation of the configurational entropy is assumed decoupled from ensemble relaxation and follows its path to the equilibrium state. The quickness of the change of the configurational entropy is assumed proportional to the magnitude of $S\left(t\right)-\tilde{S}\left(\xi \left(t\right)\right)$ and inversely proportional to the relaxation time

(2) $$\frac{dS\left(t\right)}{dt}=-\frac{S\left(t\right)-\tilde{S}\;\left(\xi \left(t\right)\right)}{\tau \left(\xi \left(t\right)\right)}.$$

This equation can be analytically solved. The solution is solved by the “variation of constants” method. The solution is the product of the varied constant, *X*, and the homogeneous solutions S*

(3) $$S\left(t\right)=S^{*}\left(t\right)X\left(t\right).$$

The solution for the homogeneous equations is

(4) $$S^{*}\left(t\right)=A\exp\left[-\int_{0}^{t}\frac{dt^{\prime }}{\tau \left(\xi \left(t^{\prime }\right)\right)}\right].$$

When inserting *S*(*t*) from Equation (3) into Equation (2) and expressing S*(*t*) by Equation (4), then an equation for *X*(*t*) is obtained, namely

(5) $$\frac{dX\left(t\right)}{dt}=\frac{\tilde{S}\left(\xi \left(t\right)\right)}{S^{*}\left(t\right)\tau \left(\xi \left(t\right)\right)},$$

yielding the explicit solution

(6) $$X\left(t\right)=B+\int_{0}^{t}\frac{\tilde{S}\left(\xi \left(t\right)\right)}{S^{*}\left(t\right)\tau \left(\xi \left(t\right)\right)}dt^{\prime }.$$

Hence, the solution for Equation (2) is

(7) $$S\left(t\right)=A\left\{B+\int_{0}^{t}\tilde{S}\left(\xi \left(t^{\prime }\right)\right)\frac{\exp\left[-\int_{0}^{t^{\prime }}\frac{dt^{\prime \prime }}{\tau \left(\xi \left(t^{\prime \prime }\right)\right)}\right]}{\alpha \tau \left(\xi \left(t^{\prime }\right)\right)}dt^{\prime }\right\}\exp\left[-\int_{0}^{t}\frac{dt^{\prime }}{\tau \left(\xi \left(t^{\prime }\right)\right)}\right].$$

Imposing the initial condition *S*(0) = *S_i_* the solution of Equation (2) is

(8) $$S\left(t\right)=S_{i}\exp\left[-\int_{0}^{t}\frac{dt^{\prime }}{\tau \left(\xi \left(t^{\prime }\right)\right)}\right]+\int_{0}^{t}\tilde{S}\left(\xi \left(t^{\prime }\right)\right)\frac{\exp\left[-\int_{t^{\prime }}^{t}\frac{dt^{\prime \prime }}{\tau \left(\xi \left(t^{\prime \prime }\right)\right)}\right]}{\tau \left(\xi \left(t^{\prime }\right)\right)}dt^{\prime }.$$

Relaxation time must be given to evaluate the entropy relaxation. A simple Debye dependence on temperature is assumed for the sake of simplicity

(9) $$\tau \left(T\right)=\tau_{0}\exp\frac{\alpha }{T}=\tau_{0}\exp\left[\frac{T_{g}}{T}\ln\left(\frac{\tau (T_{g})}{\tau_{0}}\right)\right],$$

where *τ*_0_ is the relaxation time at infinite temperature and $\tau (T_{g})$ is the relaxation time at glass transition temperature *T_g_*. In further calculations, we use $\tau_{0}=10^{-14}s$ and *τ*(*T_g_*) = 100 s. The first is suggested by Angell [16] and the second is conventionally used for the definition of glass transition temperature [17,18]. It can be deduced that fixing the relaxation times at infinity and the glass transition temperatures follow Angell’s approach for the classification of glass by fragility [19]. On the other hand, *τ*_0_ value is not universally accepted [20]. In the glass industry or practice, viscosity is used for the determination of *T_g_*. Although it is based on the convention, viscosity 10^12^ Pa s is commonly used. The viscosity and relaxation time are coupled by the Maxwell equation by the shear modulus at infinite frequency. The modulus is not constant for all materials; therefore, if *T_g_* is determined from viscosity, usage of variation modulus (standard value is 10^10^ Pa) leads to the variation of relaxation times differing from 100 s.

**Figure 1:**
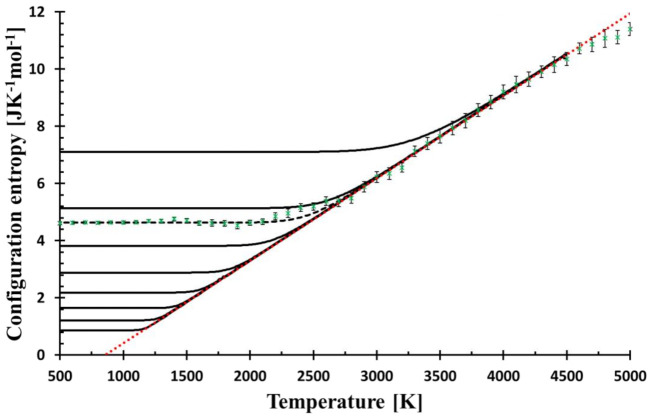
The temperature dependence of the configurational entropy shows how it changes with the cooling rates (from top to bottom, the curves correspond to *f* = 3 × 10^9^, 3 × 10^7^, 6.3 × 10^6^, 3 × 10^5^, 3 × 10^3^, 3 × 10^1^, 3 × 10^−1^, 3 × 10^−3^, 3 × 10^−5^ Ks^−1^) as calculated by Equation (8). Green crosses represent the configurational entropy evaluated from MD snapshots; the error bars (2σ) come from the snapshot statistics. MD values are best fitted by the cooling rate *f* = 6.3 × 10^6^ Ks^−1^ (broken black line). The red dotted bar is the linear fit (*R*^2^ = 0.998) of MD data in the temperature interval of 2700–4600 K, the line is used for the calculation of relaxation, see Equation (10). The mol unit refers to mol of SiO_2_.

The suggested relaxation temperature dependence is experimentally observed for viscosity in the limited temperature range above *T_g_* and corresponds to the ideal strong glass. Vitreous silica is an archetypal strong glass so that this dependence should be a good starting point for the description of the relaxation of configurational entropy. To perform the calculation for silica glass, *T_g_* was set to 1450 K. Finally, the temperature dependence of $\tilde{S}$ is needed and is taken from the equilibrium part of MD simulation, specifically from the linear part of the configurational entropy curve. For the linear fit of MD yields, see Figure 1.

(10) $$\tilde{S}\left(T\right)=-2.463+0.002882T.$$

The only explicit time-dependent parameter left is the temperature *T*, which changes linearly with time. *T* = *T*_0_ − *f t*, where *f* is the cooling rate with *T*_0_ = 4500 K, and $S_{i}\left(t=0\right)=\tilde{S}\left(4500\;K\right)=10.506$. Equation (10) introduces the temperature *T^K^* = 855 K, Kauzmann temperature [21], at which the configurational entropy reaches zero. This temperature corresponds to the hypothetical glass transition temperature of infinitely slowly cooled glass.

## 3. Results

### MD Configurational Entropy versus Relaxation Model

The results of the relaxation of the configurational entropy for various cooling rates, *f*, given by calculation as described in *Entropy relaxation* are presented in Figure 1. Simultaneously, the configurational entropy evaluated from MD simulations is shown for comparison. The lower cooling rate shifts the bending part of the curve commonly taken as the glass transition region to the lower temperature. The cooling rate with the best match of the low-temperature MD configurational entropy (the constant part) is equal to *f* = 6.3 × 10^6^ Ks^−1^, which is a value many orders lower than the effective cooling rate used for cooling of the simulated system, 10^13^ Ks^−1^.

**Figure 2:**
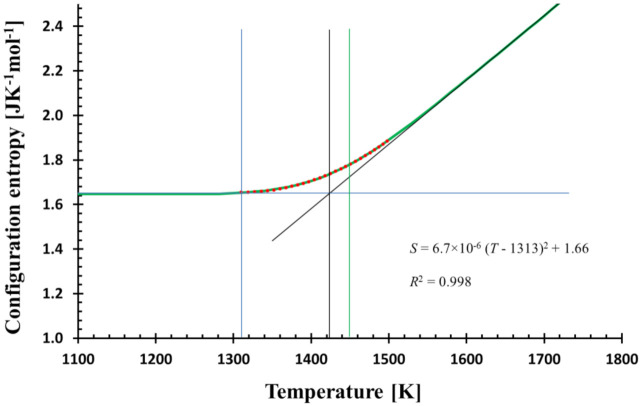
The temperature dependence of the calculated configurational entropy (green bold line) and its quadratic fit (red dots) in the glass transition region (1310–1500 K) shows the match; the equation of the fit together with *R*^2^ is shown. The quadratic fit determines the temperature T* (blue vertical line) where the structure is frozen-in. The calculation was performed with the cooling rate *f* = 0.3333 Ks^−1^. Tool temperature (vertical black line) and glass transition temperature corresponding to 100 s of relaxation time (green vertical line) are also shown.

The glass transition region is the transitional region between two lines: low- and high-temperature courses. It has been shown previously [9] that MD configurational entropy can be fitted in this region by the quadratic function of temperature

(11) $$S\left(T\right)=S_{0}+\beta {\left(T-T^{*}\right)}^{2},$$

where *β* was interpreted as the quickness of structural changes and T* as freezing-in temperature. However, no physical explanation justifying this quadratic shape was provided. Because of the harmony between the relaxation model and MD data, it is no surprise that the model curve fits the quadratic function in this region as well. Figure 2 shows a perfect match between the relaxation model with the experimentally recommended cooling rate (0.3333 Ks^−1^) [20] and the assumed functional shape. The shape of the configurational entropy in the glass transition region and the temperature T* (thin blue line in Figure 2), can then be understood within the relaxation model. Moreover, Figure 1 and Figure 2 manifest that the relaxation of the configurational entropy in MD simulation is principally Debye-like, and the quadratic shape of the relaxation in the glass transition region is a consequence of this relaxation. The closeness of experimental *T_g_* and *T_g_* as given by the model extrapolated down to the experimental cooling rate suggests the plausibility of the model to real glasses.

Equation (11) suggests the interpretation of *S*_0_ as residual entropy. The value 1.66 JK^−1^ mol^−1^ (mol of SiO_2_) is, however, at about three-times lower than the experimentally determined one [22]. On the other hand, MD simulation provides a value in harmony with the experiment. The discrepancy could be related to parametrization of MD potentials as all results should be attributed to the “MD glass” and not to real glass.

## 4. Discussion

**Figure 3:**
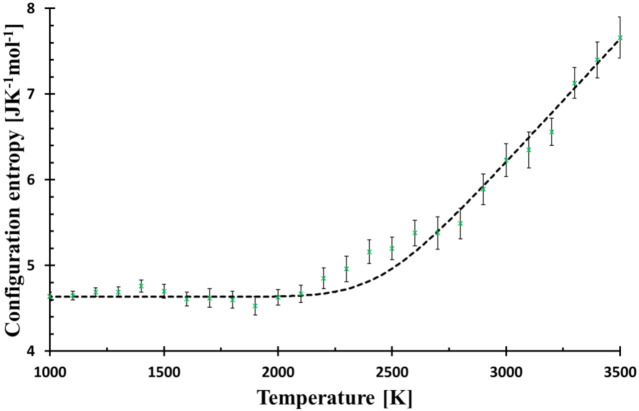
Configurational entropy calculated by Equation (8) (black broken line) for *f* = 6.3 × 10^6^ Ks^−1^ and that evaluated from MD simulation (green crosses). The Figure is zoomed from Figure 1 to better demonstrate the deviations of MD from Debye relaxation in the glass transition region.

Molecular dynamics mimics the formation of glass and enables the study of glass transition in diminutive details. However, it is questionable how close to reality the simulation is. The most challenging region of the glass-melt system is the glass transition, where the relaxation is empirically described by a stretched exponential. The stretching is rationalised by the presence of a set of various processes or the existence of structural motives with different relaxation times [23]. The current approach uses just one relaxation time, even though it is temperature and system dependent. It is not in contradiction with the concept of hierarchical relaxation times [23]. First, the studied system is an archetype of the strong glass that shows a higher similarity to the Arrhenius curve in the comparison with the fragile glasses. Second, the calculated configuration entropy is based on the ring distribution; therefore, it does not cover all possible relaxations. Third, it is still MD glass, so it must be compared with a real glass with great caution. Fourth, there is not a perfect harmony in the glass transition region (see Figure 3), so other types of relaxation could play an important role here.

Glass structure can be described by various descriptors, among them, the Radial distribution function is the most common due to its availability by some spectroscopic methods. On the other hand, the configurational entropy, a fundamental quantity parametrizes the glass transition by a simple, single-value descriptor of the structure with an obvious advantage of its explicit position in thermodynamics (part of Gibbs energy). The approach based on basic rings utilises the decomposition of the simulated structure into unambiguously determined structural pieces with a great benefit of preserving the medium-range order. As the used method is rather heuristic and based on the implicit assumption that the smaller rings are energetically more stable, it must be confronted with experiments. Previous papers showed that the suggested method is in qualitative harmony with the behaviour of the melt undergoing solidification or heated glass turning into a melt [11]. The model also exhibits the delayed relaxation when the system was reheated with the higher rate than it had been cooled [8]. The lowering of *T_g_* with a decreasing cooling rate was also confirmed. Nevertheless, each comparison with experimental data must be completed on alert while keeping in mind that it is MD glass studied and not real glass. The curve of configurational entropy versus temperature numerically fits a quadratic function in the glass transition region with a surprising precision; T* was understood as the structure freezing-in temperature [9]. Nevertheless, such dependence was not supported by any model. Therefore, the simple Debye relaxation of configurational entropy was assumed (1) to compare the expected deviation from MD simulation, (2) to reduce the number of adjustable parameters, and (3) have a simple and straightforward interpretation of the findings. In Figure 3, the MD system approaching the transition region reveals a slightly slower relaxation compared to the Debye relaxation (“bump” at around 2300 K), but with a further decrease in temperature, its relaxation is accelerated and matches the calculated curve again. Additional decrease in temperature (relaxation time prolongation) shows other small “deviation-waves” indicating the presence of some structural feedback or coupling of configurational entropy with another quantity. The use of the stretched exponential cannot describe the observed “damped wave” behaviour of the system in the transformation region.

MD relaxation of configurational entropy is the best matched with Debye relaxation with a cooling rate *f* = 6.3 × 10^6^ Ks^−1^, a value significantly higher than the recommended experimental rate but also much lower than the effective MD cooling rate. At this value, the internal change of the relaxation time is comparable with the quickness of the temperature change. Although the calculation coincides with the simulation, the transferability of the model to the experimental cooling rate is questionable. Nevertheless, the calculation (bear in mind, the temperature dependence of configurational entropy is still based on MD simulation, see Equation (10)) generates the transition region with *T_g_* differing only by 10 K from the experimental one. Thus, supporting the plausibility of the performed extrapolation into the experimentally used cooling rates. On the other hand, the discrepancy between experimental residual entropy and the model entropy cannot be overlooked.

The quadratic fit perfectly matches the calculated and MD configurational entropies in the glass transition region. From a mathematical point of view, it is a consequence of the (practically) zero first derivative at and below some temperature T*, where the structure is frozen. Here, T* = 1313 K (see Figure 2). If the calculated configurational entropy is used with Adam–Gibbs [24] or MYEGA [25] relation for viscosity, no singularity is generated; T* determines only the temperature below which viscosity is practically constant.

## 5. Conclusions

The glass structure obtained by computer simulation was transferred into the undirected graph from which the configurational entropy was calculated. The linear part of the configurational entropy versus temperature curve was taken as an input for the theoretical calculation of the Debye relaxation of the configurational entropy. It was shown that the relaxation of the configurational entropy of MD glass in the glass transition range corresponds to the effective cooling rate of 6.3 × 10^6^ Ks^−1^, which is significantly lower than the MD effective cooling rate. Extrapolation of results to the experimentally recommended cooling rate yields the glass transition temperature close to the experimental one. Despite a nearly perfect agreement between MD configurational entropy temperature course and the Debye relaxation model, some deviations can be found in the glass transition region.
